# Effect of Red-to-Near Infrared Light and a Nitric Oxide Donor on the Oxygen Consumption of Isolated Cytochrome c Oxidase

**DOI:** 10.1089/photob.2020.4978

**Published:** 2021-07-01

**Authors:** Brendan Quirk, Harry T. Whelan

**Affiliations:** Department of Neurology, Medical College of Wisconsin, Milwaukee, Wisconsin, USA.

**Keywords:** photobiomodulation, cytochrome c oxidase, oxygen consumption

## Abstract

**Objective:**

To study the effects of 670 and 830 nm irradiation on oxygen consumption by cytochrome c oxidase (CCO) in a Clark electrode type reaction chamber. To explore the effect of irradiation on the nitric oxide (NO) donor-induced inhibition of oxygen consumption.

**Background:**

Most theories of photobiomodulation (PBM) involve the enzyme CCO as a cellular target for red-to-near infrared light (R-NIR) irradiation. Attempts to measure the effect of irradiation on the kinetics of CCO have failed to demonstrate a significant effect. It remains to explore the effects of irradiation on the consumption of oxygen. NO has been proposed as a possible mediator for PBM due to its inhibitory effects on CCO. Studying the effect of R-NIR on NO-induced inhibition of oxygen consumption is needed to explore this thesis.

**Methods:**

Oxygen consumption assays at 22°C were performed in a Mitocell MT200A system equipped with a 1302 oxygen electrode. R-NIR irradiation at 670 nm (41 mW/cm^2^) or 830 nm (31 mW/cm^2^) was provided to the reaction mixture. Calculated second-order rate constants were compared with control runs at four cytochrome c concentrations. Assays were also performed with or without NO donor and/or light for two substrate concentrations.

**Results:**

Kinetics constants for oxygen consumption with or without R-NIR showed no significant differences with either wavelength at any substrate concentration. The NO donor showed significant inhibition that was not relieved by irradiation.

**Conclusions:**

This lack of effect by R-NIR calls into question both the CCO activity model and the NO inhibition relief model of PBM.

## Introduction

The study of photobiomodulation (PBM) by red-to-near infrared light (R-NIR) has now had a long torturous history. There is no longer a need to list the clinical and preclinical areas in which it has been studied; any number of extensive reviews have been published. What has always been lacking is basic mechanistic studies with the aim of discovering what processes truly lie at the heart of the phenomenon we call PBM.^1,2^ Although most theories of PBM involved the mitochondrial electron transport chain enzyme cytochrome c oxidase (CCO) as a cellular target for light irradiation, when we first initiated our basic science work in this area, only one study of R-NIR irradiation of CCO had been published.^3^ This study found minor effects on the kinetics of the reactions of CCO by 632 nm irradiation.

Our attempt to confirm and extend this study, however, failed to support these results when measuring the kinetics of CCO.^4^ Our results, while calling into question the CCO activity model of R-NIR PBM, still left open the possibility of an effect on the other half of the two-substrate CCO reaction: the consumption of oxygen. Therefore, this study was conceived as a follow-on study to fill this gap, studying oxygen consumption effects in a Clark electrode type reaction chamber with 670 and 830 nm light.

Nitric oxide (NO) has traditionally been proposed as a possible mediator for PBM due to its inhibitory effects on CCO that may be relievable by the application of R-NIR. A more recent role for NO has been proposed as a light-induced product of nitrite reduction by CCO.^5^ These ideas have been covered in some detail in our recent review.^2^ In this study, we also tested the NO inhibition relief theory of PBM using an NO donor Spermine NONOate and the oxygen consumption apparatus.

## Materials and Methods

CCO was prepared from bovine heart according to the method of Soulimane and Buse.^6^ Protein concentrations were determined by the DC Protein Assay (Bio-Rad, Hercules, CA). CCO concentrations were calculated using a molecular mass of 204,696.^6^ The preparation was stored in small aliquots at −80°C in 10 mM Tris-HCl, 300 mM NaCl, and 0.1% Triton X-100, pH 7.6. Cytochrome c was prepared fresh for each experiment by dissolving ∼3 mg/mL cytochrome c in a buffer containing 0.1 M 2-(N-morpholino)ethanesulfonic acid (MES) and 10 μM ethylenediaminetetraacetic acid (EDTA), pH 6.0. Concentrations of stock solutions were determined spectrophotometrically at 550 nm using a milimolar extinction coefficient^4^ of ɛ_550ox_ = 8.60 ± 0.15/(L·cm).

**FIG. 1.**
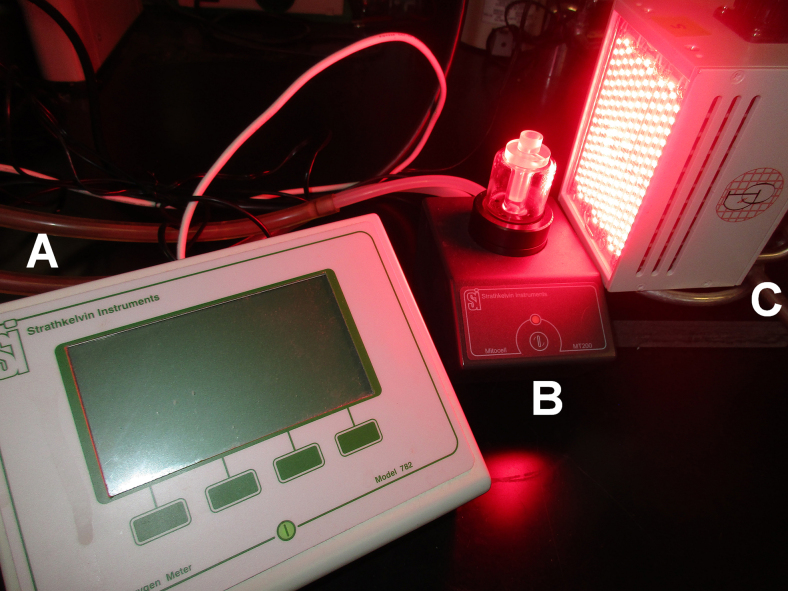
Photograph of experimental apparatus: **(A)** Strathkelvin 782 dual channel oxygen meter; **(B)** Mitocell MT200A with 300 μL stirred reaction chamber, plastic spacer/plunger with injection port, Clark type 1302 oxygen electrode, and surrounding water jacket; and **(C)** Quantum Devices 670 or 830 nm light-emitting diode (LED) array with control box/power supply.

**FIG. 2.**
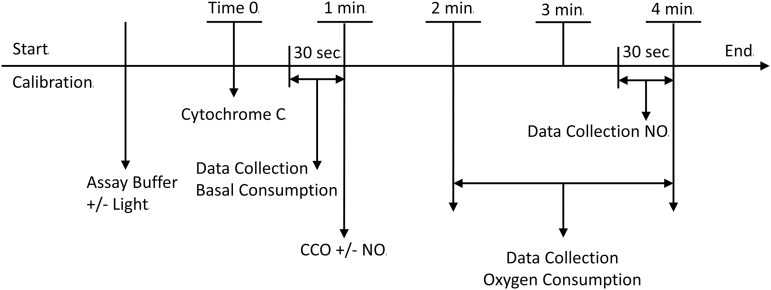
Timeline of individual replicate. Preparation: calibration with oxygen saturated water, addition of assay buffer, and initiation of irradiation if used. Time 0: addition of cytochrome c. 30–60 sec: data collection for basal oxygen consumption. 60 sec: addition of CCO and/or NO. 120–240 sec: data collection for oxygen consumption without NO. 210–240 sec: data collection for oxygen consumption with NO. CCO, cytochrome c oxidase; NO, nitric oxide.

Oxygen consumption assays (under nonphysiological conditions to facilitate comparison with earlier studies^3,4^) at 22°C (using a thermostated circulating water bath) were performed in a Mitocell MT200A Strathkelvin 782 2-channel oxygen system (Warner Instruments, Hamden, CT) equipped with a 1302 oxygen electrode using a fluorinated ethylene propylene membrane. Figure 1 shows a schematic of the experimental setup. Before each experiment, the electrode was calibrated at 0 oxygen concentration by using 2% sodium sulfite, and at 267 μM oxygen using air saturated water. Before each run, the electrode was again calibrated to 267 μM oxygen. The assay buffer consisted of 0.1 MES, 10 μM EDTA, 0.1% n-Dodecyl β-D-maltoside (#D4641; Sigma-Aldrich), 2.5 mM ascorbic acid, and 0.5 mM N,N,N′,N′-tetramethyl-p-phenylenediamine dihydrochloride (TMPD, #87890; Sigma-Aldrich) at pH 6.0. The open assay buffer container was agitated on high using a vortex mixer for 1 sec to ensure uniform oxygen saturation, 320 μL was placed in the 300 μL reaction chamber (the excess volume allows for capillary action to fill the injection port and forms a seal against oxygen from the atmosphere), sealed, and stirring commenced. Cytochrome c substrate in assay buffer was then introduced, and oxygen readings were obtained for 1 min. The reaction was initiated by adding appropriate amounts of CCO solution and followed for 3 min. Each assay was performed in triplicate at six CCO concentrations per cytochrome c concentration. All runs were performed in randomized order to prevent time-order bias. See Fig. 2 for a timeline of an individual replicate.

Exposure to NIR was achieved by mounting a 670 or 830 nm light-emitting diode (LED) array (Quantum Devices Inc., Barneveld, WI) flat against the glass reaction chamber and operating at full power throughout the run. The irradiance measured in the center of the reaction chamber was 41 mW/cm^2^ for 670 nm and 31 mW/cm^2^ for 830 nm. The reactions were run with the room lights on. The irradiance from the room lights reaching the reaction mixture was measured at 0.05 mW/cm^2^, or <0.2% of the applied NIR irradiation. As these are continuous, real-time, exposures, and measurements, fluence, or dosage, is neither calculated nor relevant. Irradiance measurements were performed with a photodiode calibrated by Quantum Devices.

Observed first-order rate constants (k_obs_) were calculated from the slope of a plot of ln[O_2_] versus time. Rates for basal oxygen consumption were calculated using the last 30 sec of data from the cytochrome c only assay segment, and rates for the gross CCO oxygen consumption were calculated from the last 2 min of the CCO assay segment. Rates for net CCO oxygen consumption were calculated by subtracting the basal rates from the CCO segment rates. Second-order rate constants (k′) were calculated from plots of k_obs_ versus [CCO] using a weighted linear least squares analysis (PSI-Plot; Poly Software International, Pearl River, NY), and converted to turnover number (TN) by multiplying by the saturating substrate (oxygen) concentration. Statistical significance was tested using a two-tailed Student's *t*-test, with significance set at *p* < 0.05.

Oxygen consumption assays for NO inhibition studies were performed in a similar manner, substituting 0.1 M 3-(N-morpholino)propanesulfonic acid at pH 7.4 for the MES. An NO donor was prepared by dissolving 1 mg Spermine NONOate (#82150; Cayman Chemical, Ann Arbor, MI) in 1 mL 0.01 M NaOH. For NO runs, 3 μL fresh NO donor was added immediately after the CCO was added, for an initial NO donor concentration of 38.1 μM. To allow for the fact that NO generation is not linear, only the last 30 sec of the CCO oxygen consumption segment was used for data analysis.

## Results

**FIG. 3.**
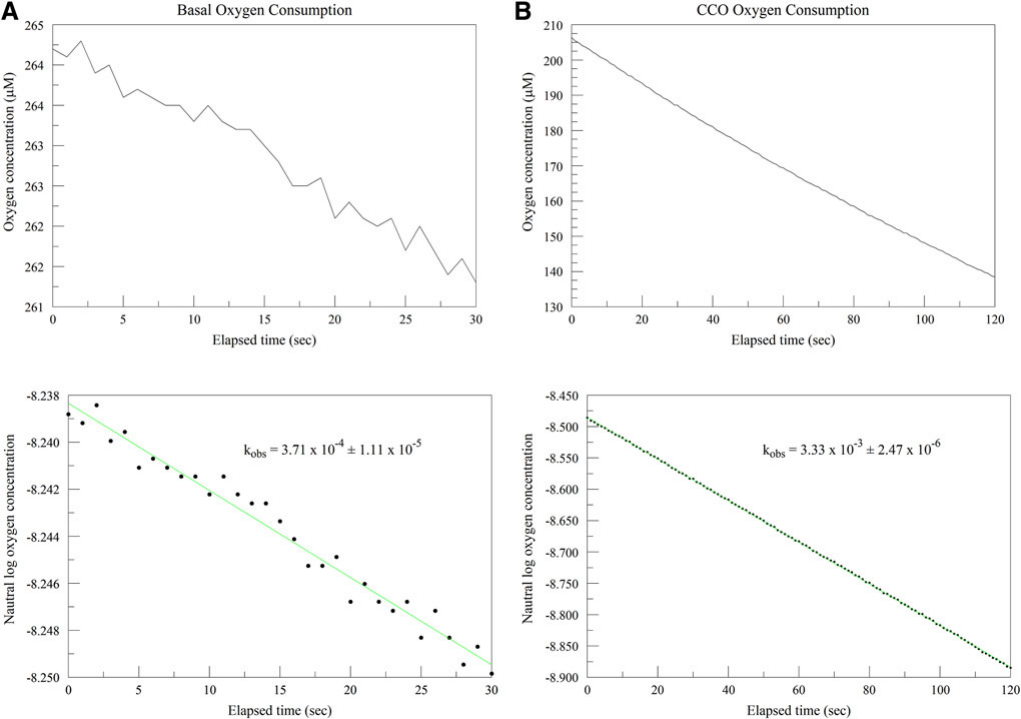
Typical oxygen consumption assay. **(A)** Buffer/cytochrome c segment. *Top* figure is oxygen concentration versus time; *bottom* figure, log plot determining kinetic constant (k_obs_). **(B)** Buffer/cytochrome c/CCO segment. *Top* and *bottom* figures as panel **(A)**.

Figure 3 illustrates segments from a typical oxygen consumption assay. Panel A is from the buffer/cytochrome c only segment, showing basal oxygen consumption for the system. The top figure is the oxygen concentration in the system versus time, while the bottom figure illustrates how the observed kinetic constant (k_obs_) is determined. In this system, cytochrome c is maintained in a predominately reduced, and constant, condition by the reducing activity of the excess ascorbate. Oxygen consumption then follows a pseudofirst-order process, and k_obs_ is determined by the slope of a plot of ln[oxygen] versus time.

Panel B shows the buffer/cytochrome c/CCO segment in the same manner. The oxygen consumption in this case is fast enough that a curved trace is seen, as is proper for a pseudofirst-order process. Data analyses that use a straight line fitted to the oxygen trace are, therefore, only approximations and are not accurate except for slow consumption over a very short time period. The bottom figure illustrates how the log plot yields a straight line and allows calculation of k_obs_.

The k_obs_ for net oxygen consumption for CCO can be calculated by subtracting basal consumption k_obs_ from gross consumption k_obs_ in the CCO segment of the run.^7^ Initial TN can then be obtained by multiplying k′ by saturating oxygen concentration.

**FIG. 4.**
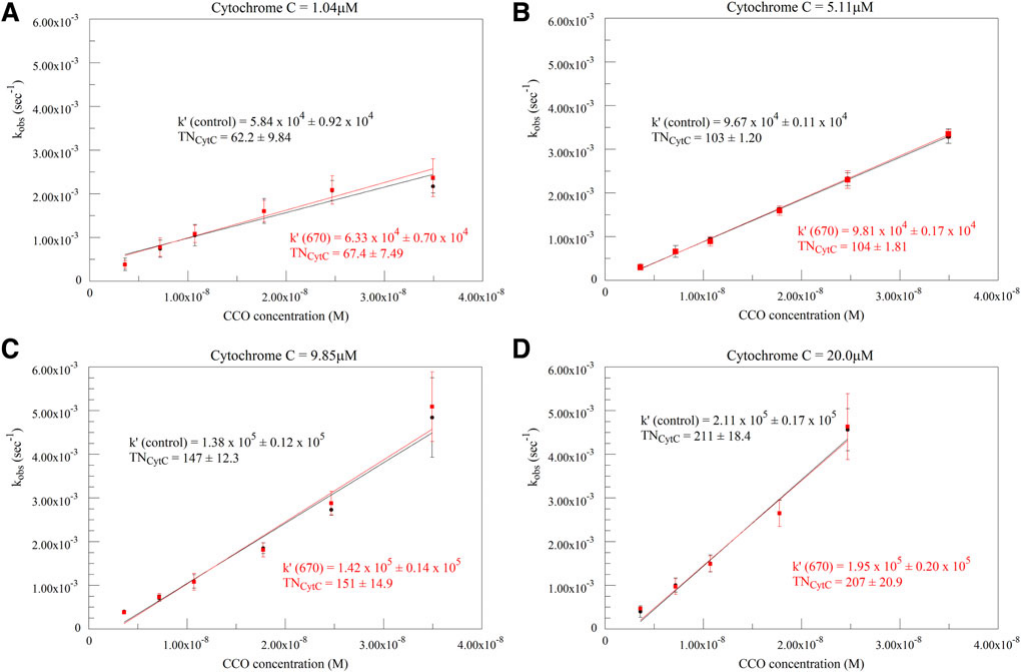
Calculated k_obs_ versus CCO concentration at 1 **(A)**, 5 **(B)**, 10 **(C)**, and 20 **(D)** μM cytochrome c. Black traces represent control runs, red traces represent the corresponding 670 nm irradiated runs. Second-order rate constants (k′) and TN expressed in electron equivalents obtained from the slopes are given on the plots. TN, turnover number.

Plots of calculated k_obs_ versus CCO concentration at four different cytochrome c concentrations are presented in Fig. 4. The second-order rate constant k′ can be obtained from the slope of these plots. TN is expressed in electron equivalents and represents the number of cytochrome c molecules oxidized per CCO molecule in 1 sec at saturating oxygen concentration. Actual oxygen consumption is one-fourth of this, as four electrons are needed to reduce one oxygen molecule. Expressing TN in this manner facilitates comparing TN across a variety of studies and experimental protocols. In these plots, the control (black) and 670 nm irradiation (red figures) values can be seen to be nearly identical at each cytochrome c concentration. The k′ and TN values in each case show no significant differences.

**FIG. 5.**
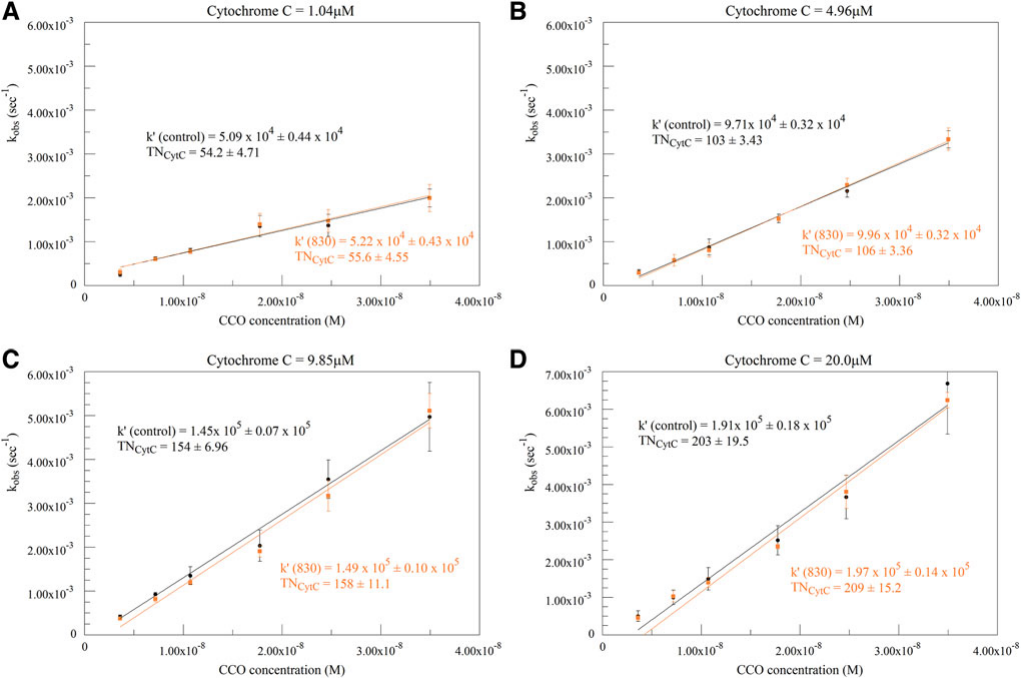
Calculated k_obs_ versus CCO concentration at 1 **(A)**, 5 **(B)**, 10 **(C)**, and 20 **(D)** μM cytochrome c. Black traces represent control runs, orange traces represent the corresponding 830 nm irradiated runs. Second-order rate constants (k′) and TN expressed in electron equivalents obtained from the slopes are given on the plots.

Similar plots for 830 nm irradiation (orange figures) are shown in Fig. 5. Again, k′ and TN values show no significant differences.

**FIG. 6.**
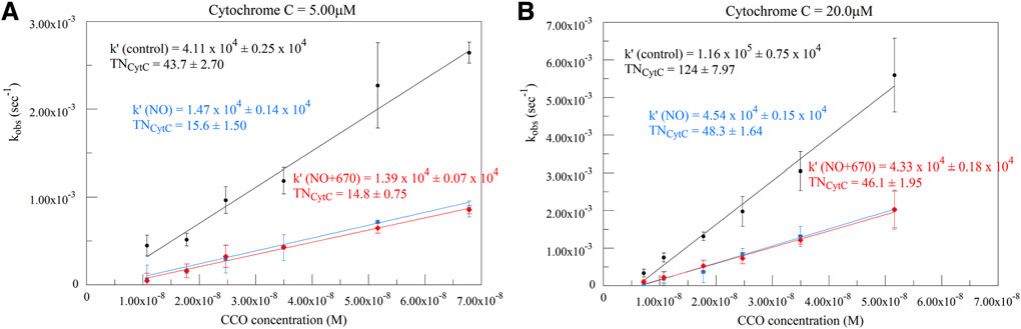
Calculated k_obs_ versus CCO concentration at 5 **(A)** and 20 **(B)** μM cytochrome c. Black traces represent control runs, blue traces represent the corresponding NO donor runs, and red traces represent NO donor plus 670 nm irradiation runs. Second-order rate constants (k′) and TN expressed in electron equivalents obtained from the slopes are given on the plots.

**FIG. 7.**
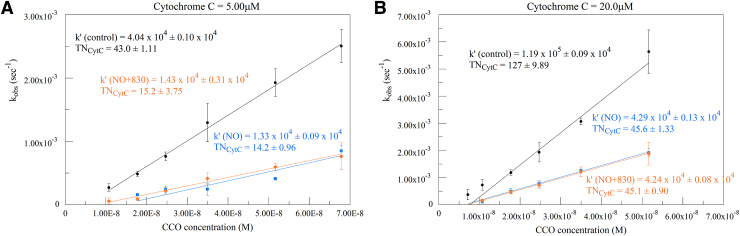
Calculated k_obs_ versus CCO concentration at 5 **(A)** and 20 **(B)** μM cytochrome c. Black traces represent control runs, blue traces represent the corresponding NO donor runs, and orange traces represent NO donor plus 830 nm irradiation runs. Second-order rate constants (k′) and TN expressed in electron equivalents obtained from the slopes are given on the plots.

Plots for experiments in the presence of the NO donor Spermine NONOate are shown in Fig. 6 (670 nm irradiation) and Fig. 7 (830 nm). Results from control runs are shown in black figures, NO donor runs in blue figures, and irradiated runs with NO donor in red or orange figures. The presence of NO clearly has a strong inhibiting effect on oxygen consumption. The addition of R-NIR irradiation, however, has no effect on relieving this inhibition.

## Discussion

This study is essentially a follow-on to a previous study^4^ exploring the effect of R-NIR on the oxidation of cytochrome c by CCO. In that study, preirradiation of isolated CCO by 670 or 830 nm light had no effect on the kinetic parameters. In addition, concurrent irradiation of the reaction mixture by 660 nm light also had no effect. This is in contrast to a study by Pastore et al.^3^ showing a small positive effect of 632 nm irradiation with low cytochrome c/CCO ratios, and a small negative effect with high ratios. The same pattern was observed for oxygen consumption as for CCO. In this study, we took up the second half of the two-substrate reaction, and measured oxygen consumption with or without 670 or 830 nm irradiation.

Figures 4 and 5 clearly show that, in our hands, the consumption of oxygen in a sealed Clark electrode type system is not affected by irradiation at the two wavelengths tested. Although this could be expected from our results with cytochrome c, we felt that it should be explored since the oxygen consumption protocol does not use precisely the same reaction system as the CCO protocol.^7^ In the cytochrome c protocol, oxygen is in excess and remains constant, whereas the oxidation state of the cytochrome c changes during the reaction. In the oxygen protocol, the cytochrome c is maintained in a mostly reduced state by a combination of ascorbate and TMPD, while oxygen levels decrease. In addition, in the oxygen protocol, the cytochrome c remains associated with the CCO molecule, and electron transfer proceeds to the bound cytochrome c. In the cytochrome c protocol, the reduced cytochrome c must bind to the CCO, transfer an electron, and diffuse away before the next catalytic cycle.^7^ This difference in mechanisms impairs the ability to compare results between different studies. Nevertheless, the TNs determined in this study are similar to our previous values, although not showing the plateauing at high cytochrome c levels. Indeed, the TNs seen are in a range, 50–210, that is consistent with typical values seen in other studies.^8^

Pastore's values^3^ for TNs are similar to ours, whereas PBM effects range from a 21% increase at low substrate concentrations to a 15% decrease at high substrate concentrations. Since these effects are small, experimental artifacts are more likely, hence our attempt to confirm them. At this point, we can only speculate that undetermined differences in experimental methods or enzyme preparation and handling are the causes of these discrepancies.

As laid out in a recent review,^2^ there have been strong reasons to consider light energy relief of NO inhibition as a prime candidate for the underlying mechanism behind PBM. In this theory, rather than an acceleration of the kinetic cycle, irradiation promotes mitochondrial energy production by relieving the inhibition of CCO by bound NO. It has been shown that NO rapidly and reversibly inhibits steady-state turnover of CCO and is competitive with oxygen.^9,10^ Further, the NO binds at the active site heme a_3_, and is white light labile.^11^ Clearly, oxygen consumption studies with or without NO and irradiation could address this question.

Figures 6 and 7 show inhibition by the NO donor Spermine NONOate. However, there is no effect on this inhibition by 670 or 830 nm irradiation. The kinetic values determined for the NO-irradiated runs are not significantly different from the NO-only runs, whereas both sets of NO and NO-irradiated runs are significantly lower than controls.

**FIG. 8.**
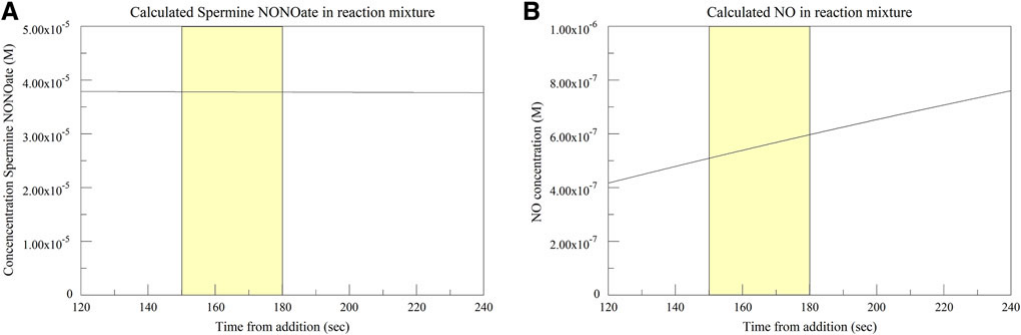
Modeling of NO production and consumption in reaction system. **(A)** Calculated NO donor remaining; yellow area represents time period used in kinetics determination. **(B)** Calculated NO in system at same time period.

The NO used in this study is provided by the breakdown of an NO donor Spermine NONOate. This molecule is stable at high pH, but degrades at low or neutral pH, providing two moles of NO per mole donor. The half-life for this process is 230 min at 22–25°C and a pH of 7.4 (Cayman product literature). In aqueous solution, NO oxidizes relatively quickly to nitrite, with a half-life of 7.4 min.^12^ Using standard first-order kinetics calculations, the remaining NO donor and NO in solution can be modeled throughout the reaction course. In Fig. 8, the remaining donor during the data collection period of 150–180 sec after donor addition (210–240 sec after start of run) is relatively stable at 37.8 μM (panel A). The calculated NO in solution, augmented by donor degradation and decreased by NO oxidation, is more variable, at roughly 500–600 nM (panel B). Although variable, the NO level is stable enough in this case to explore the effects of R-NIR on CCO inhibition. The roughly 60–70% inhibition seen in these experiments is not inconsistent with the 270 nM Ki for NO measured by Brown and Cooper.^9^

We have seen, to date, no report of an attempt to demonstrate relief of NO inhibition of CCO by R-NIR on isolated enzyme. Borutaite et al.,^13^ in 2000, did report relief of NO inhibition on mitochondrial CCO activity when irradiated with either white or 425 nm light. She states that there is no obvious physiological relevance of this in animals; one may assume since light of these wavelengths does not penetrate beyond the very surface of animal tissues.

**FIG. 9.**
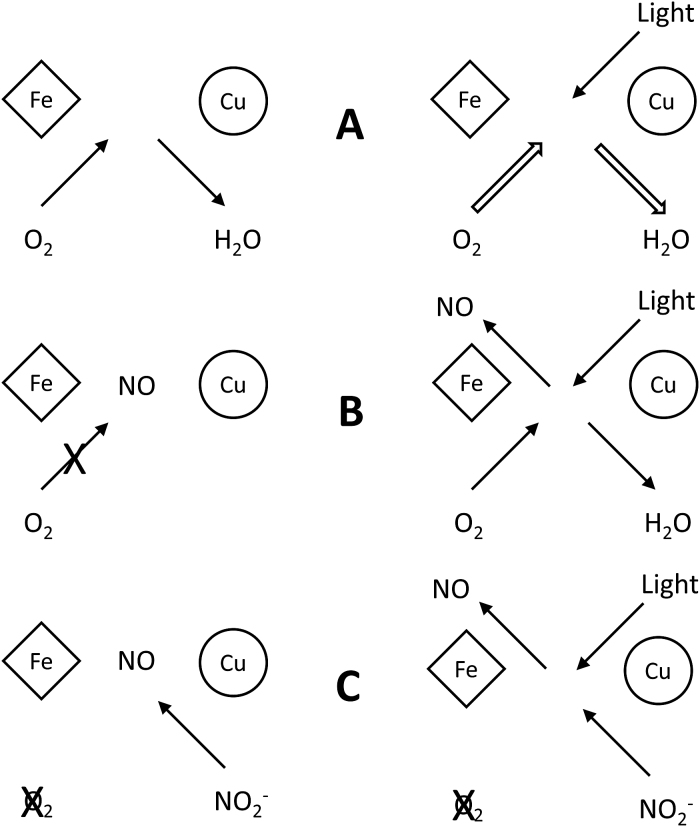
Schematic of some proposed PBM mechanisms. **(A)** Reduction of oxygen to water at active site of CCO. Light absorbed by CCO in some manner accelerates this reaction. **(B)** Competitive inhibition by NO at the active site reduces oxygen binding, hence slows reaction rates. Light causes removal of NO and resumption of normal rates. **(C)** In a low oxygen environment, CCO reduces endogenous nitrite to NO, which can act in various downstream and signaling mechanisms. Light enhances NO off rate, increasing cytoprotective NO production. PBM, photobiomodulation.

Although this study was concerned primarily with detecting changes in oxygen consumption due to irradiation with R-NIR, it does not address any proposed PBM mechanisms that do not manifest themselves in this manner. Figure 9 shows two theories of PBM that are addressed by this study: acceleration of oxygen reduction and relief of NO inhibition. Also illustrated is an alternative theory, enhancement of NO production from nitrite. The authors believe that there currently is no truly reliable and repeatable PBM phenomenon demonstratable at the mechanistic level. There is an enormous mass of reported epiphenomenon, under every conceivable experimental condition and protocol, that never clearly resolves itself into a reliable structure that may point the way to the truth of PBM. Until we can demonstrate such phenomenon, accessible to and repeatable by any group involved in the field, progress toward understanding PBM will remain stymied.

## Future Directions

We propose to study further the effect of light on NO production by CCO. The importance of NO is already established, and others are doing a comprehensive job of studying the role of NO in metabolism and signaling. The following are some possibilities:

1. Calculate NO production and CCO activity rates and kinetic constants. Previous studies have been illustrative but not quantitative. Reliable numbers will be important to application of PBM.
2. Study the equilibrium binding of NO to CCO to probe the idea that NO production enhancement of light is by improving NO off-rate from the active site.
3. Expand convincing experiments showing NO production by CCO^5^ to include Mb and Hb. NO release from these appears to be essential to cardioprotection.^14^
4. Do a thorough survey of PBM light parameters for any proven and reproducible light effects. If optimum wavelengths can be reliably and narrowly located, compare with spectral properties of turnover intermediates to further mechanistic information related to PBM.

## Conclusions

The consumption of oxygen by CCO has not been shown to be affected by R-NIR irradiation at 670 or 830 nm applied concurrently with the enzymatic assays. This lack of effect by R-NIR, in conjunction with earlier study with CCO, further calls into question the CCO activity model of R-NIR PBM. In addition, R-NIR does not relieve CCO inhibition by NO generated from an NO donor, also calling into question the NO-inhibition relief model of PBM. These experiments were not conducted under physiological conditions however, so at this point, the relevance to underlying mechanisms of PBM *in vivo* is, therefore, limited. Further study under physiological conditions and on the possible light-induced generation of NO by CCO, along with downstream signaling and metabolic effects, appears to be the next step in this journey.
